# Ultrasound Assessment of the Third Ventricle Diameter: Agreement With CT Results in the Pediatric Population

**DOI:** 10.1097/CCE.0000000000001387

**Published:** 2026-03-09

**Authors:** Safae Dehbi, Zhor Zeghari, Chaimae Es-Sebbani, Badr Ettouhami, Siham El Haddad, Redouane Abouqal, Aziza Bentalha, Salma Ech Cherif El Kettani

**Affiliations:** 1 Pediatric Intensive Care Unit (PICU) of the Children’s Hospital of Rabat, IBN SINA University Hospital, Rabat, Morocco.; 2 MOHAMMED V University, Rabat, Morocco.; 3 Laboratory of Biostatistics, Epidemiology and Clinical Research, Faculty of Medicine and Pharmacy, Rabat, Morocco.; 4 Radiology Department of the Children’s Hospital of Rabat, IBN SINA University Hospital, Rabat, Morocco.

**Keywords:** critical care, pediatrics, third ventricle, tomography, ultrasonography

## Abstract

**OBJECTIVES:**

To evaluate the agreement between transcranial ultrasound (TUS) and CT measurements of third ventricle diameter in critically ill children.

**DESIGN:**

Prospective observational cohort study.

**SETTING:**

PICU of the Children’s Hospital of Rabat, Morocco.

**PATIENTS:**

Children 1 month to 15 years old admitted with acute neurologic distress who underwent brain CT.

**INTERVENTIONS:**

TUS was performed within 1 hour of CT using a phased-array probe through bilateral transtemporal windows.

**MEASUREMENTS AND MAIN RESULTS:**

Of 150 screened patients, 148 were included (median age 5 yr; 68% male). Feasibility was high, with bilateral acoustic windows obtained in 98.6% of cases. The median third ventricle diameter was 3.6 mm by both CT and TUS. Bland-Altman analysis demonstrated good agreement between TUS and CT: mean bias 0.11 mm (limits of agreement, –0.78 to 1.01 mm) on the right side and 0.16 mm (–0.81 to 1.13 mm) on the left. Agreement was highest for smaller third ventricle diameters, which accounted for most measurements. No patients had a midline shift or had undergone decompressive craniectomy at the time of imaging.

**CONCLUSIONS:**

TUS provides reproducible bedside measurements of third ventricle diameter in children, with good agreement compared with CT in patients without midline shift or major surgical alterations. While not a substitute for comprehensive neuroimaging, TUS may serve as a nonirradiating adjunct for serial ventricular monitoring, particularly for monitoring for the development of hydrocephalus and during external ventricular drain management. Further research should evaluate interoperator reproducibility, applicability in patients with mass effect, and integration into clinical workflows.

**KEY POINTS**

**Question**: Does transcranial ultrasound provide third ventricle diameter measurements that agree with CT in critically ill children?

**Findings**: In this prospective observational cohort study of 148 children, third ventricle diameter measured by transcranial ultrasound showed good agreement with CT, with minimal bias and narrow limits of agreement. Median third ventricle diameter was identical by both methods, and agreement was statistically significant across the observed measurement range.

**Meaning**: Transcranial ultrasound can serve as a reliable, nonirradiating bedside adjunct for monitoring third ventricle size in selected pediatric neurocritical care patients.

**RESEARCH IN CONTEXT**

- In adults, transcranial ultrasound (TUS) can estimate third ventricle diameter and has been shown to correlate with CT and MRI. Pediatric data are limited, particularly after fontanelle closure when standard cranial ultrasound windows are no longer available.
- In this prospective cohort of 148 critically ill children, TUS through the temporal window showed good agreement with CT for third ventricle diameter. Success was high, with nearly all patients insonated, although interoperator generalizability remains unestablished.
- Findings cannot be generalized to children with midline shift, decompressive craniectomy, or marked ventriculomegaly, as these were not represented in our cohort. Interoperator reliability was not assessed, and measurements were performed by a single-trained intensivist.

**AT THE BEDSIDE**

- In most children, including those with traumatic brain injury or status epilepticus, bilateral acoustic temporal windows were obtainable, supporting the use of TUS for bedside assessment.
- TUS should not be interpreted as a substitute for CT or MRI. Instead, it may be useful for monitoring trends in third ventricle size, such as during external ventricular drain weaning or monitoring for the development of hydrocephalus, where repeated nonirradiating measurements are valuable.
- Broader studies are needed to define normative values indexed to age and head size, assess reproducibility across operators, and determine clinical thresholds that can guide management decisions.

CT and MRI provide comprehensive anatomical information for the evaluation of acute neurologic disorders in critically ill children. However, repeated imaging is limited by radiation exposure, the need for patient transport, and sedation requirements. Consequently, bedside neuromonitoring tools that allow safe and repeatable assessment are of particular interest in pediatric neurocritical care. Today, transcranial ultrasound (TUS) is an accessible, nonirradiating diagnostic tool with a favorable learning curve, allowing repeated use at the patient’s bedside (1). These qualities make TUS particularly valuable in situations where frequent monitoring is necessary or transportation is risky. Unlike transcranial Doppler (TCD), which measures cerebral blood flow velocities, TUS provides direct visualization of intracranial structures, including linear measurement of the third ventricle.

TCD is already recommended for neuromonitoring in cases of severe traumatic brain injury (TBI) (2), subarachnoid hemorrhage (3), and acute ischemic stroke (4), where it helps detect vasospasm, impaired autoregulation, and prognostic indicators. Building on this, TUS complements these techniques by enabling visualization of intracranial structures, notably the third ventricle, as first described by Bogdahn et al in 1990 (5). Since then, several adult studies have compared TUS measurements of the third ventricle with CT or MRI (5, 6), confirming its feasibility and usefulness for monitoring for the development of hydrocephalus. These findings indicate that TUS could serve as a valuable bedside adjunct to conventional imaging, especially for repeated assessments.

However, data on children are limited (7). In pediatric patients, cranial ultrasound is widely used to detect brain conditions such as intraventricular hemorrhage or hydrocephalus. Still, conventional approaches usually rely on fontanelle windows and might not effectively capture deeper ventricular structures once the fontanelles close (8). Repeated CT scans after fontanelle closure expose children to ionizing radiation and often require sedation, whereas assessment of the third ventricle through the preserved temporal window offers a radiation-free, bedside, and repeatable method for ongoing ventricular monitoring (9). Importantly, recent pediatric studies, including Kerscher et al (7), have shown that TUS measurements of the third ventricle can reliably reflect ventricular dynamics and may assist in the detection and follow-up of hydrocephalus. A prior study from our institution by El Adioui et al (10) evaluated TUS for the assessment of hydrocephalus in children and demonstrated a relationship between third ventricle measurements obtained by TUS and CT. That work provided important preliminary evidence supporting the feasibility of this technique. The present study builds on this prior institutional experience by including a larger pediatric cohort and applying agreement analysis to compare third ventricle measurements obtained by TUS and CT.

Nonetheless, the clinical significance of a single linear measurement remains uncertain, especially because it is not indexed to head or patient size, and there are no universally accepted reference values. This underscores the need for further research to establish normative ranges and to find the best ways to incorporate these measurements into clinical decision-making. Therefore, this study aims to evaluate the agreement between third ventricle diameter measured by TUS and CT in critically ill children and to assess the feasibility of routine bedside use.

## METHODS

We conducted a prospective observational study at the PICU of the Children’s Hospital of Rabat. Data were collected from October 2024 to May 2025. All children between 1 month and 15 years old admitted to the PICU or emergency department for neurologic distress and who had a brain CT scan were eligible for enrollment. The exclusion criteria included: inability to perform TUS within 1 hour of brain CT scan, patients with inadequate acoustic temporal windows, and neonates, who in our institution are cared for in a separate neonatal ICU and not admitted to the PICU, where this study was conducted. In neonates, ventricular visualization is usually achieved through the anterior fontanelle rather than the temporal acoustic window, and therefore, they were outside the scope of our study.

At admission, patient information such as age, sex, weight (kg), height (cm), and body mass index was recorded. The patient’s medical history, indication for the CT scan, and the presence or absence of external ventricular drainage were also documented. TUS measurements of the third ventricle diameter were performed within 1 hour of the brain CT scan to minimize any changes in ventricular status. All measurements were carried out and verified by a single experienced intensivist with formal training in point-of-care ultrasound and over 5 years of clinical experience in pediatric critical care, who was blinded to the brain CT measurements.

### Transcranial Ultrasound Measurements

**Figure 1.**
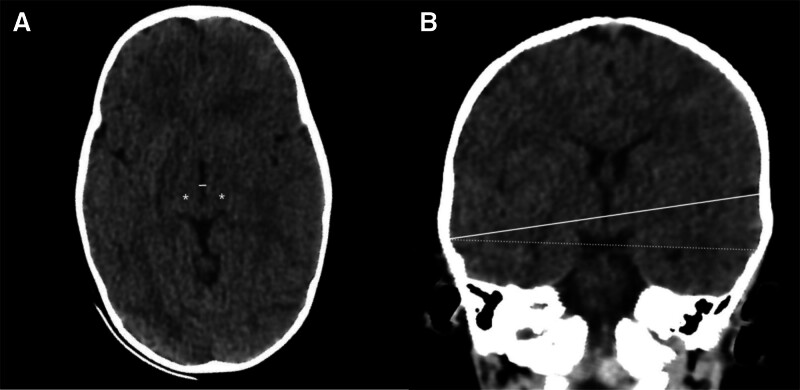
Brain CT images of an 11-mo-old boy admitted with status epilepticus. **A**, Axial CT section at the level of the thalami (*asterisks*) showing the third ventricle with caliper placement (*white line*). **B**, Coronal CT reconstruction illustrating the orientation of the ultrasound probe for third ventricle assessment, with the reference plane shown as a *dotted line* and the measurement axis as a *solid line*.

**Figure 2.**
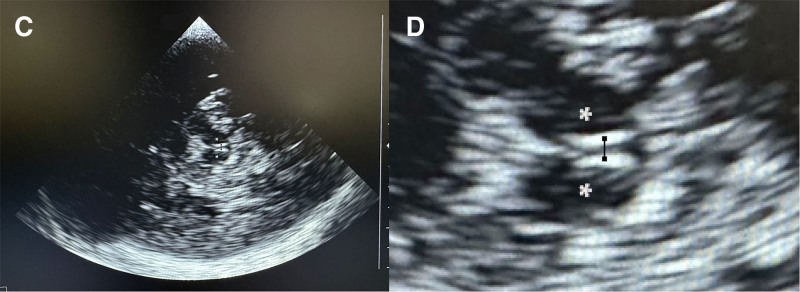
Transcranial ultrasound assessment of the third ventricle in the same patient. **A**, Insonation through the temporal acoustic window demonstrating the hyperechogenic margins of the third ventricle, surrounded by the hypoechogenic thalami (*asterisks*). **B**, Zoomed view of the third ventricle highlighting precise caliper placement between the echogenic ventricular walls.

The measurement was performed on sedated children in a supine position, head up 30°, using a Sonosite Edge 2 ultrasound with a low-frequency echocardiography probe (5-1 MHz). The TCD preset available on the device was selected to optimize acoustic penetration through the temporal bone window. The latter was located just above the zygomatic arch and anterior to the external auditory canal, as described in previous TUS methodology studies (11). The third ventricle diameter was measured on both sides. First, the mesencephalon was tracked with a classic “butterfly-wing” structure, then the probe was moved 10° upward to locate the third ventricle with its hyperechogenic margins, the surrounding hypoechogenic thalami, and the posterior hyperechogenic pineal gland (**Figs. 1** and **2**; and **Supplemental Fig. 1**, https://links.lww.com/CCX/B607). To improve visualization of the third ventricle, the image was electronically zoomed to focus on the ventricular margins. Measurements were then taken in the axial plane between the hyperechogenic ventricular walls using the system’s electronic calipers. Figures 1 and 2 and Supplemental Figure 1 (https://links.lww.com/CCX/B607) show the probe orientation and caliper placement.

### Brain CT Measurement

A CT scan was performed using a 16-slice CT scanner with helical acquisition. The slice thickness was 0.6 mm, and a multiplanar reconstruction was obtained. The third ventricle diameter was calculated independently by a radiologist who was blinded to the TUS measurements and clinical data. The measurement was done using an axial section through the middle of the thalamus; two measurements were taken and averaged.

### Statistical Analysis

A descriptive analysis was performed. Categorical variables are expressed in numbers and frequencies, and continuous variables are expressed in mean (sd) if the distribution was Gaussian or in median (interquartile range [IQR]) if not. The agreement between the TUS and CT scan was assessed using the Bland-Altman plot. The normality of the measurement differences was verified, confirming that the assumptions of Bland-Altman analysis were met. The mean difference between measurements, upper and lower limits of agreement, and corresponding two-sided 95% CIs were obtained.

Clinically acceptable limits of agreement were predefined at ± 1.5 mm, based on previously published Bland-Altman analyses. In adults, Widehem et al (5) reported limits of agreement between TUS and CT measurements ranging from –2.7 to 3.3 mm, indicating that a ± 1.5 mm threshold reflects clinically acceptable precision. In a large pediatric cohort, ultrasonographic third ventricle diameter measurements compared with MRI demonstrated a range of limits of agreement of –0.99 to 1.18 mm (7). We therefore selected ± 1.5 mm as a pragmatic threshold likely to reflect measurement equivalence without clinical impact, cognizant that future studies should establish age- and size-adjusted thresholds. Statistical analyses were conducted using the free-access JAMOVI software, Version 2.6.24 (Jamovi project, Sydney, Australia).

### Ethics

Ethics approval was obtained from the Faculty of Medicine of Rabat’s Ethics Committee for Biomedical Research (reference number CERB 143-24; approval date: September 25, 2024). All procedures involving human participants were conducted in accordance with the ethical standards of the institutional research committee and the 1975 Declaration of Helsinki and its subsequent amendments. Written informed consent was obtained from the parents or legal guardians of all participants. Assent was obtained whenever possible from children who were sufficiently conscious and of an appropriate age to give it. The findings from this study are reported in accordance with the Guidelines for Reporting Reliability and Agreement Studies.

## RESULTS

During the study period, 150 patients were screened for eligibility. Two patients were excluded due to inadequate acoustic windows, resulting in 148 patients included in the final analysis. All included patients had bilateral acoustic windows and underwent a single TUS examination. Patients with CT-confirmed midline shift (*n* = 8) were excluded at screening because ventricular distortion and clinical urgency precluded the standardized TUS assessment outlined in the study protocol.

**Table 1** presents patient demographics and clinical characteristics. Patients ranged in age from 1 month to 15 years (median 5 yr), and 102 (68%) were male. The most frequent admission diagnoses were TBI and status epilepticus. Because patients may have multiple medical or surgical conditions, categories are not mutually exclusive, and percentages are not expected to sum to 100%. All TBI patients were classified as moderate to severe cases according to the Glasgow Coma Scale (moderate: 9–12, severe: ≤ 8), as defined in international guidelines (12), and all required PICU admission. No patient had a midline shift at the time of imaging, and none underwent a decompressive craniectomy. Surgical wounds did not interfere with temporal window acquisition. Continuous electroencephalogram monitoring is not available in our unit; thus, electrode placement did not impact feasibility.

**TABLE 1.** Demographic and Medical Patient Characteristics

Variable	Value (*n* = 148)
Age^a^ (yr)	5 (1.5–10.0)
Gender^b^ (male)	102 (68)
Patient’s history	
Hydrocephalus^b^	11 (7)
Epilepsy^b^	35 (23)
Brain tumor^b^	6 (4)
Admission cause	
Traumatic brain injury^b^	77 (51)
Status epilepticus^b^	47 (31)
Coma^b^	7 (4)
Meningitis^b^	17 (11)
ICU admission^b^	45 (30)
Presence of an external ventricular drainage^b^	1 (0)
Presence of ventriculoperitoneal shunt^b^	0 (0)

a Data are presented as median (interquartile range).

b Data are presented as *n* (%).

The median average of the third ventricle diameter measured by the CT scan using an axial section was 3.6 mm (IQR, 3.1–4.2 mm). The median of the third ventricle diameter measured by TUS was 3.6 mm (IQR, 3.0–4.2 mm) for the right side and 3.6 mm (IQR, 3.0–4.2 mm) for the left side (**Supplemental Table 1**, https://links.lww.com/CCX/B607).

**Figure 3.**
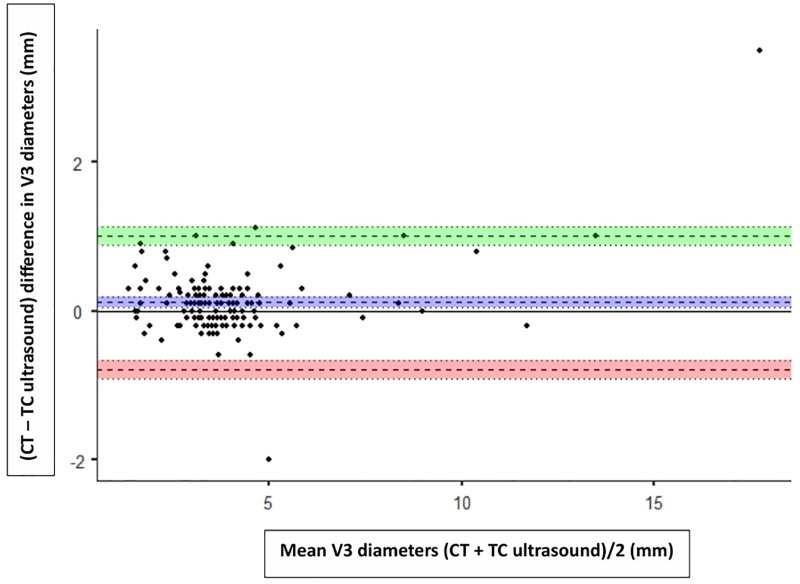
*Bland-Altman plot* showing agreement between transcranial (TC) ultrasound (TUS; left temporal acoustic window) and brain CT scan for third ventricle (V3) diameter measurements. The *dashed blue line* represents the mean bias, while the *dashed green* and *red lines* indicate the upper and lower limits of agreement, respectively. Mean = mean V3 diameter, calculated as (CT + TUS)/2 (mm). Difference = difference in V3 diameter, calculated as (CT–TUS; mm).

**Figure 4.**
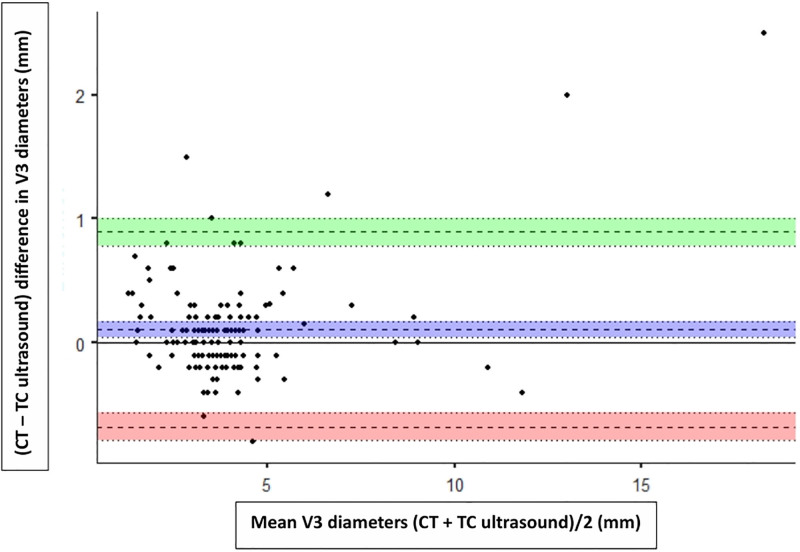
*Bland-Altman plot* showing agreement between transcranial (TC) ultrasound (TUS; right temporal acoustic window) and brain CT scan for third ventricle (V3) diameter measurements. The *dashed blue line* represents the mean bias, while the *dashed green* and *red lines* indicate the upper and lower limits of agreement, respectively. Mean = mean V3 diameter, calculated as (CT + TUS)/2 (mm). Difference = difference in V3 diameter, calculated as (CT–TUS; mm).

To determine the agreement between TUS and CT scan measurements, we used Bland-Altman plots. The mean bias between CT and TUS was 0.11 mm (95% CI, 0.04–0.19 mm), with limits of agreement ranging from –0.78 mm (95% CI, –0.90 to –0.65 mm) to 1.01 mm (95% CI, 0.88–1.14 mm) on the right side. On the left side, the mean bias was 0.16 mm (95% CI, 0.08–0.24 mm), and the limits of agreement ranged from –0.81 mm (95% CI, –0.95 to –0.67 mm) to 1.13 mm (95% CI, 0.99–1.27 mm). Additionally, the Bland-Altman plots demonstrated strong agreement across the observed range of values. Since the vast majority of our patients had third ventricle diameters less than 10 mm (median, 3.6 mm; IQR, 3.0–4.2 mm), agreement is best interpreted within this range (**Figs. 3** and **4**).

## DISCUSSION

To our knowledge, this study represents the largest pediatric cohort to date in which third ventricle diameter measurements by TUS were directly compared with CT scan. The key finding is the good agreement between the two modalities, with only minimal systematic bias, suggesting that TUS can provide reproducible bedside estimates of third ventricle size. Procedural success was excellent, with 98.6% of patients having both acoustic temporal windows, although generalizability to multiple operators was not assessed. The thinness of the skull bone in younger children provides greater accessibility compared with adults, thereby improving the visualization of intracranial structures (7).

The Bland-Altman plot was used in our study to assess the agreement between TUS and CT measurements of the third ventricle. Results showed that over 97.33% of values were within the limits of agreement, with low bias on both sides. Our findings are supported by Kerscher et al (9), who reported a mean bias of 0.09 ± 0.55 mm (mean ± sd) between TUS and MRI/CT measurements of the third ventricle diameter in a pediatric population.

However, few studies in the current literature directly compare these two techniques, and those that do are limited to adult populations. For instance, Schminke et al (13) assessed the transverse diameter of the third ventricle in 27 adults with multiple sclerosis using both TUS and MRI. They reported a mean bias of 0.35 mm (95% CI, 0.17–0.87 mm), with Bland-Altman limits of agreement ranging from –2.23 mm to 2.93 mm. In a larger study, Widehem et al (5) evaluated 100 adult patients in a neurocritical care setting. The mean difference between CT and TUS was 0.36 ± 1.52 mm on the right side, with limits of agreement of –2.7 and 3.3 mm, and 0.25 ± 1.47 mm on the left side, with limits of agreement of –2.7 and 3.1 mm.

Importantly, our data were concentrated in smaller third ventricle diameters, with the majority below 10 mm, which corresponds to the range most commonly encountered in our PICU. Agreement between TUS and CT was tightest in this range, likely because smaller ventricles are less affected by the angulated insonation used in TUS (10–15° upward transverse) compared with the axial plane of CT. As the third ventricle diameter increases, the angulated TUS measurements tend to slightly overestimate CT measurements, leading to greater bias. Clinically, this finding suggests that TUS is most useful as a screening and trend-monitoring tool in children with normal or mildly enlarged ventricles, rather than as a definitive diagnostic modality in advanced ventriculomegaly. Agreement deteriorates as ventricular diameter increases, underscoring that TUS should prompt confirmatory cross-sectional imaging when marked dilation is suspected. This pattern is consistent with Kerscher et al (7), who reported that significant deviations in TUS measurements were generally observed only when third ventricle diameters exceeded 20 mm.

We observed slightly different biases by side, with a slight edge for the left acoustic temporal window. Several factors may contribute, including the modest upward insonation angle used in TUS vs. strictly axial CT sections. Importantly, all measurements were performed by a single experienced operator, so side-dependent differences may be influenced by proceduralist technique. None of the patients in our cohort had radiographic evidence of midline shift or underwent decompressive craniectomy, and our findings cannot be generalized to populations with these factors. Future work should incorporate systematic documentation of shift, surgical status, and multiple operators to assess how they influence agreement and laterality.

The practical axial resolution of a 5–1 MHz phased-array probe is typically on the order of 0.5–1 mm in soft tissue, as higher-frequency probes (7–18 MHz) are required for submillimetric precision, particularly in superficial imaging contexts (14). While our Bland-Altman analysis demonstrated narrow mean biases, the true precision of TUS is likely wider, constrained by these probe limitations and real-world factors such as beam steering and operator-dependent factors. Furthermore, the third ventricle’s shape changes with distention, adjacent structure atrophy, or pathology, complicating the assumption of a uniform, two-plane structure and thereby limiting the reliability of a single linear measurement. While the nonirradiating, repeatable nature of TUS is advantageous at the bedside, a single linear third ventricle measurement should be interpreted as one component of a broader clinical assessment rather than a standalone determinant of management. The measurement is not indexed to head size or age in our study, and it cannot, on its own, characterize regional ventricular asymmetries or extra-axial pathology. Accordingly, we view TUS as a monitoring adjunct for trend detection (e.g., hydrocephalus surveillance or external ventricular drain weaning) rather than a replacement for comprehensive CT/MRI when broader diagnostic information is required (5). Beyond third ventricle assessment, TUS has been described in neurocritical care as an accessible bedside tool—sometimes referred to as a “brain stethoscope”—that allows nonirradiative evaluation of brain structures and cerebral blood flow (15). Prior studies have reported applications including bedside hematoma assessment, evaluation of midline shift, and hydrocephalus monitoring (16–18). Importantly, the ability to avoid patient transport to the CT scanner may reduce risks such as worsening intracranial hypertension or disconnection of external ventricular drains. In particular, TUS may support management of external ventricular drains: during weaning, recurrent hydrocephalus can lead to a rapid increase in third ventricle diameter and clinical deterioration, which may be difficult to recognize in unconscious patients. In this context, TUS offers a safe, repeatable bedside screening method that can complement clinical evaluation by providing timely detection of ventricular enlargement (5).

A major limitation of our study is that we did not assess intraobserver and interobserver reliability, as the study was not designed to do so. As a result, further data collection is necessary to evaluate the impact of operator-related factors on the reproducibility of TUS measurements, independent of patient characteristics. We therefore recommend that intensivists undergo structured training programs to improve skill acquisition and enhance measurement consistency. In our cohort, two older children (> 12 yr) were excluded due to inadequate acoustic windows, a finding that aligns with pediatric data showing that approximately 9–10% of children may not be assessable via a transtemporal approach (11) and with reports that increased temporal squama thickness reduces feasibility, particularly in older children and adults (19). Another important limitation is that our data were concentrated in smaller third ventricle diameters, with agreement being tightest below 10 mm, the range most commonly encountered in our PICU. Consequently, our results should be interpreted as applicable primarily within this spectrum, and further studies that include children with more pronounced ventriculomegaly are needed to evaluate agreement across the full range of ventricular sizes. Finally, although our cohort included patients with TBI and malignancy, none demonstrated midline shift or had undergone decompressive craniectomy. Thus, our findings cannot be generalized to populations where mass effect or major surgical alterations significantly distort ventricular anatomy, and future studies should address these contexts specifically.

## CONCLUSIONS

This study highlights the potential role of TUS as a bedside tool in pediatric neurocritical care. We found good agreement between TUS and CT for measuring third ventricle diameter, supporting its feasibility in PICU settings. Rather than serving as a replacement for CT, TUS may provide a reproducible method for trend detection, particularly in the context of hydrocephalus surveillance and external ventricular drain management. Further studies, including broader patient populations and interoperator assessments, are needed to define its clinical impact and establish standardized protocols for integration into routine practice.

## Supplementary Material
