# Supplementary material for: Ultrasound Assessment of the Third Ventricle Diameter: Agreement With CT Results in the Pediatric Population
Source: Crit Care Explor. 2026 Mar 9;8(3):e1387. doi: 10.1097/CCE.0000000000001387 (PMC12975263; doi:10.1097/CCE.0000000000001387)
Supplement: Supplementary file 1 [file cc9-8-e1387-s001.pdf]

Supplemental material

Title page

- Supplemental Figure 1: Probe placement for obtaining transcranial ultrasound (TUS) images in an 11-month-old boy admitted to the PICU for status epilepticus.
- Supplemental Table 1: Third ventricle measurement using CT scan and TUS.

Table of Contents

Materials	Page number
Supplemental Figure 1	2
Supplemental Table 1	3

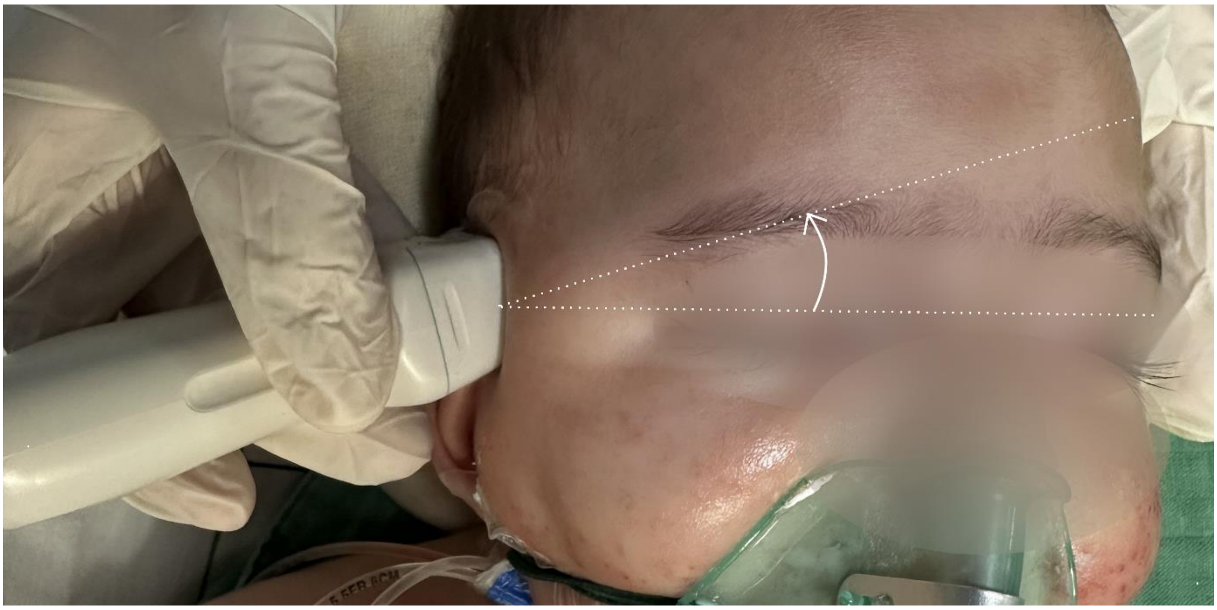

Supplemental Figure 1: Probe placement for obtaining transcranial ultrasound (TUS) images in an 11-month-old boy admitted to the PICU for status epilepticus.

Supplemental Table 1: Third ventricle measurement using CT scan and TUS

Variable	Value (N = 148)
CT scan measurement [a] (mm)	3.6 [3.1-4.2]
TUS measurement: right side [a] (mm)	3.6 [3.0–4.2]
TUS measurement: left side [a] (mm)	3.6 [3.0–4.2]

a: Data are in median (Interquartile Range)
